# Public health in Vietnam: scientific evidence for policy changes and interventions

**DOI:** 10.3402/gha.v6i0.20443

**Published:** 2013-02-25

**Authors:** Nguyen Duc Hinh, Hoang Van Minh

**Affiliations:** 1 Institute for Preventive Medicine and Public Health, Center for Health System Research, Hanoi Medical University, Hanoi, Vietnam; 2 Department of Health Economics, Institute for Preventive Medicine and Public Health, Center for Health System Research, Hanoi Medical University, Hanoi, Vietnam

Vietnam has made impressive progress toward improving the health status of the population, with progress that equals or surpasses that of many neighboring countries. Life expectancy in Vietnam is 72.8 years (70.2 for men and 75.6 for women), a level that is considerably higher than that in many countries with similar levels of GDP per capita. From 1990 to 2009, the infant mortality rate fell from 44.4% to 16.0%, the under-five mortality rate dropped from 58.0% to 24.5%, and the maternal mortality ratio declined from 233 to 69 maternal deaths per 100,000 live births. Estimated to be around 18% in 2010, the rate of under-five malnutrition has also fallen dramatically. These improvements are attributable to a widespread health care delivery network, increasing numbers of qualified health workers, and expanding national public health programs (1, 2).

Although many significant achievements have been made, Vietnam's health care system still faces many difficulties and challenges. Recent health sector reviews have identified a number of health issues in this regard. These include an emerging a double burden of noncommunicable diseases (cardiovascular diseases, cancer, diabetes, etc.) and infectious diseases (HIV/AIDS, H1A1, etc.), an ageing population, inadequate capacity of the health system, and problems of inequities in access to health and health care (1–6). The findings from this cluster of papers provide further insights into today's health issues in Vietnam.

The fact that infectious diseases remain a public health concern is illustrated by Toan et al. in a study demonstrating that Dengue fever remains pervasive and that the geographical scope of the disease has expanded in recent years (7). Vietnam is also undergoing epidemiological transition whereby the burden of disease attributable to chronic noncommunicable conditions is rising rapidly. Minh et al.'s research demonstrates that chronic diseases are highly prevalent in rural populations and that households are more likely to face catastrophic health expenditure and impoverishment for chronic noncommunicable disorders (8). Lan et al. observe that the survival probability for breast cancer is lower in Vietnam than it is in countries with similar distributions of stage at diagnosis (9). Furthermore, a study led by Lan highlights the substantial costs of breast cancer treatment in central Vietnam, especially among patients who lack health insurance (10).

Smoking is common in Vietnam, partly due to the general population's poor knowledge of its health consequences. An et al. demonstrate that adult smokers, especially those belonging to ethnic minority groups, have low levels of knowledge regarding the harmful effects of smoking. Alcohol misuse is also a rising public health problem (11). In their study, Giang et al. report on common problems related to alcohol consumption among men in Vietnam and report that the share of total household expenditure on alcohol is remarkable, especially among poorer households (12). Diep et al. also suggest that alcohol-related harms present a serious public health problem among young and educated individuals (13). Linh et al. (14) discuss the relationship between alcohol use and road traffic accidents, and Phuong et al. (15) demonstrate the association between harmful use of alcohol and suicidal thoughts.

Vietnam is also experiencing a rapidly ageing population. It is therefore critical to have an in-depth understanding about quality of life and associated factors among elderly groups. In their study on ageing populations, Huong et al. find that quality of life among the elderly is strongly correlated with issues related to finances and economics, as well as with social relationships and familial support (16).

In the coming years, equity-oriented reform will be a major focus for the health system in Vietnam. Despite substantial achievements, there are still large health status disparities across regions and between demographic and socioeconomic groups. In this regard, Thuy et al. illustrate the extreme marginalization and distress among Vietnamese mothers whose children have disabilities. These authors identify modest levels of social capital among this population group, although relatively better mental health is also detected (17). Otherwise, Phuong et al. find a high prevalence of gender-based violence in Vietnam. This study observes that abused women are more likely than nonabused women to report contraceptive use and unintended pregnancies and that these factors are in turn associated with increased risks of induced abortion (18). In addition, Anh et al. observe the effects of unequally growing Vietnamese labor markets on migration and identify corresponding infrastructure improvements and public service needs in these areas. Analysis of migration can provide useful information for planning health and social services and for policymaking for national economic development (19).

Vietnam has over eight million people belonging to ethnic minorities, the majority of which live in remote and mountainous areas. These populations are relatively more disadvantaged in terms of socioeconomic and health status. The study by Xuan et al. finds poor hand washing with soap behaviors among schoolchildren in a multi-ethnic population of Vietnam, a potential cause of a number of health conditions (20). Human resources for health are an important building block of health system. The number of health workers in Vietnam has increased substantially over the past 10 years, but there are still severe shortages in remote and disadvantaged areas. In their study, Bach et al. find generally low levels of work-related satisfaction among of primary health care staff, particularly regarding salary and incentives, equipment, and the working environment. Predictors of job satisfaction identified by these authors include age, areas of work and expertise, professional education, location, and the sufficiency of staffing (21).

Based on the empirical evidence, all contributing authors have developed recommendations for policy changes and interventions in Vietnam. These recommendations are comprehensive and include primary, secondary and tertiary approaches, as well as policy-level interventions (7–21). Both the findings and the policy recommendations documented in these papers are highly relevant to health system stakeholders in Vietnam. The evidence is intended to help health system stakeholders, especially health policy makers and managers, to understand the implementation and impact of the policies and interventions that they introduce. The recommendations are intended to provide health system stakeholders with more options as they change or refine these measures.

Policy makers, managers, health staff and other health system stakeholders in Vietnam are committed to ensuring that all people attain a level of health that enables them to participate actively in the social and economic life of the communities in which they live. An important factor that can help the health system achieve this goal is the availability and quality of information on which decisions are based. As academics and scientists, we have conducted research to generate robust scientific evidence to support health planning and decision making in Vietnam. We hope that health system stakeholders will find this cluster of papers useful. We enthusiastically stress that scientific evidence on health is crucial for policy changes and interventions and, when the evidence is compelling, actions toward better health and health care should be taken.

*Nguyen Duc Hinh* President of the Hanoi Medical University, Hanoi Vietnam Head of the Department of Medical Ethics and Social Medicine Institute for Preventive Medicine and Public Health Center for Health System Research Hanoi Medical University, Hanoi Vietnam*Hoang Van Minh* Lecturer Department of Health Economics Institute for Preventive Medicine and Public Health Center for Health System Research Hanoi Medical University, Hanoi Vietnam
